# Differential clinical impact of chronic total occlusion revascularization based on left ventricular systolic function

**DOI:** 10.1007/s00392-020-01738-2

**Published:** 2020-09-02

**Authors:** Hyungdon Kook, Jeong Hoon Yang, Jae Young Cho, Duck Hyun Jang, Min Sun Kim, Juneyoung Lee, Seung Hun Lee, Hyung Joon Joo, Jae Hyoung Park, Soon Jun Hong, Je Sang Kim, Hyun Jong Lee, Rak Kyeong Choi, Young Jin Choi, Jin Sik Park, Young Bin Song, Jin-Ho Choi, Joo-Yong Hahn, Hyeon-Cheol Gwon, Do-Sun Lim, Seung-Hyuk Choi, Cheol Woong Yu

**Affiliations:** 1 Division of Cardiology, Department of Internal Medicine, Korea University Anam Hospital, Korea University College of Medicine, #73, Goryeodae-ro, Sungbuk-ku, Seoul, 02841 Korea; 2 Division of Cardiology, Department of Internal Medicine, Samsung Medical Center, Sungkyunkwan University College of Medicine, #81, Irwon-ro, Gangnam-gu, Seoul, 06351 Korea; 3 Department of Cardiovascular Medicine, Regional Cardiocerebrovascular Center, Wonkwang University Hospital, Iksan, Korea; 4 Department of Biostatistics, Korea University College of Medicine, Seoul, Korea; 5 Division of Cardiology, Department of Internal Medicine, Sejong General Hospital, Bucheon, Korea

**Keywords:** Chronic total occlusion, Revascularization, Left ventricular systolic dysfunction

## Abstract

**Background:**

The effect of chronic total occlusion (CTO) revascularization on survival remains controversial. Furthermore, data regarding outcome differences for CTO revascularization based on left ventricular systolic function (LVSF) are limited.

The differential outcomes from CTO revascularization in patients with preserved LVSF (PLVSF) versus reduced LVSF (RLVSF) were assessed.

**Methods:**

A total of 2,173 CTO patients were divided into either a PLVSF (*n* = 1661, Ejection fraction ≥ 50%) or RLVSF (*n* = 512, < 50%) group. Clinical outcomes were compared between successful CTO revascularization (SCR) versus optimal medical therapy (OMT) within each group. The primary endpoint was a composite of all-cause death or non-fatal myocardial infarction. Inverse probability of treatment weighting for endpoint analysis and a contrast test for comparison of survival probability differences according to LVSF were used.

**Results:**

Patients with RLVSF had a mean 37% ejection fraction (EF) and 19% had EF < 30%. The median follow-up duration was 1,138 days. Regardless of LVSF, the primary endpoint incidence was significantly lower in patients treated with SCR [RLVSF: 29.7% vs. 49.7%, hazard ratio (HR) = 0.46, 95% confidence interval (CI): 0.36–0.62, *p* < 0.0001; PLVSF 7.3% vs. 16.9%, HR = 0.68, 95% CI: 0.54–0.93, *p* = 0.0019], which was mainly driven by a reduction in cardiac death. The difference in survival probability was greater and became more pronounced over time in patients with RLVSF than with PLVSF (1-year, *p* = 0.197; 3-years, *p* = 0.048; 5-years, *p* = 0.036).

**Conclusions:**

SCR was associated with better survival benefit than OMT regardless of LVSF. The benefit was greater and became more significant over time in patients with RLVSF versus PLVSF.

**Graphic abstract:**

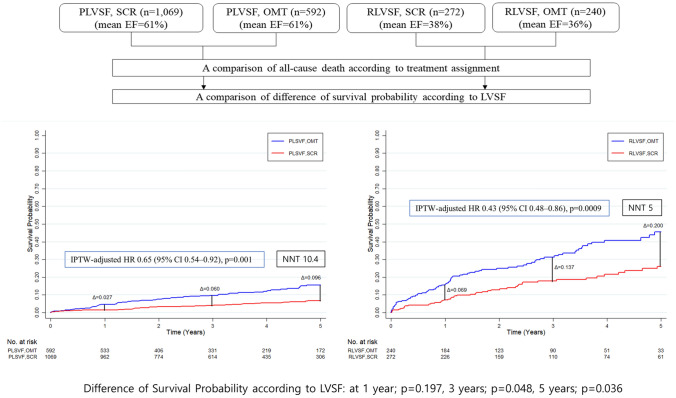

**Electronic supplementary material:**

The online version of this article (10.1007/s00392-020-01738-2) contains supplementary material, which is available to authorized users.

## Introduction

Although several limitations have been overcome through recent advances in dedicated techniques, devices, and percutaneous coronary intervention (PCI) experience [1], chronic total occlusion (CTO)-PCI still has lower success and higher complication rates compared with non-CTO-PCI [2, 3]. Therefore, PCI for CTO lesions should be considered only when the potential benefits outweigh the risk.

For non-CTO lesions, improvements in ischemia and angina from revascularization could be much greater for high-risk patients with moderate-to-severe ischemia [4]. Similarly, identification of high-risk patients for CTO revascularization might be crucial for achieving clinical benefits after CTO-PCI.

In clinical practice, many physicians hesitate to perform PCI for CTO lesions in high-risk patients with reduced left ventricular systolic function (RLVSF) because of safety concerns and uncertain benefits. There are few data regarding outcome differences after CTO revascularization based on LVSF. Therefore, the differential prognostic effects of CTO revascularization on long-term survival in preserved LVSF (PLVSF) versus RLVSF patients were investigated in this study.

## Methods

### Study population

**Fig. 1:**
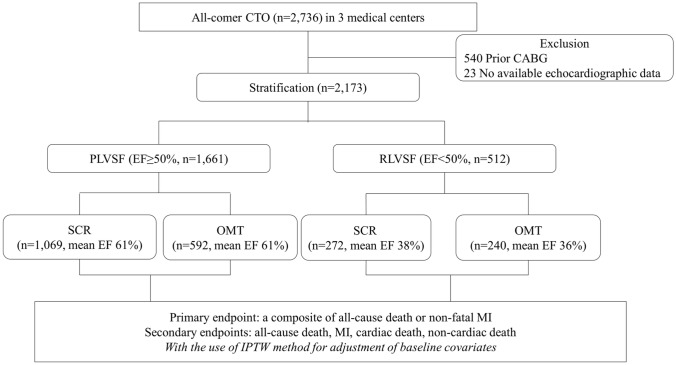
Flowchart of CTO patient stratification according to LVSF from a multicenter registry. *CABG* coronary artery bypass graft; *CTO* chronic total occlusion; *IPTW* inverse probability of treatment weighting; *LVSF* left ventricular systolic function; *MI* myocardial infarction; *OM* optimal medical therapy; *PLVSF* preserved left ventricular systolic function; *RLVSF* reduced left ventricular systolic function; *SCR* successful chronic total occlusion revascularization

A total of 2736 patients diagnosed with CTO at Korea University Anam Hospital, Sejong General Hospital, and Samsung Medical Center between March 2008 and December 2014 were reviewed. The inclusion criteria were CTO lesions detected on diagnostic coronary angiography and symptomatic angina or positive functional ischemia. Exclusion criteria were prior coronary artery bypass graft surgery or unavailable initial echocardiographic data. Patients were classified into either the PLVSF or RLVSF group based on left ventricular ejection fraction (LVEF) above (PLVSF) or below (RLVSF) 50% [5]. In each group, patients were subdivided based on treatment: successful CTO revascularization (SCR) or optimal medical therapy (OMT). The study scheme is summarized in Fig. 1. Patients received antiplatelet therapy with aspirin, and patients who underwent previous PCI or had acute coronary syndrome at diagnosis took an additional P2Y12 inhibitor for at least six months. Duration of dual antiplatelet therapy duration was determined by the attending physician. Patients received anti-anginal and heart-failure medication when appropriate. Patients were treated with statins unless contraindicated or not tolerated.

### Study endpoints and definitions

The primary endpoint was a composite of all-cause death or non-fatal myocardial infarction (MI). The secondary endpoints included all-cause death, cardiac death, non-cardiac death, and MI, which were defined according to the Academic Research Consortium (ARC)-2 classification [6]. CTO was defined as occlusion of the native coronary artery with Thrombolysis In Myocardial Infarction (TIMI) flow grade 0 for an estimated duration longer than three months [7]. Follow-up duration was defined as the interval from the first CTO diagnosis to the date of the last outpatient clinic visit or outcome of interest, whichever occurred first.

SCR was defined as final residual stenosis < 20% with TIMI flow grade 2 or 3 on the final fluoroscopic image after CTO revascularization. In patients treated with PCI, stenting or balloon angioplasty procedures were determined according to the operator’s discretion. Patients who experienced revascularization failure were included in the OMT group. Revascularization for non-CTO lesions was performed according to the physician’s discretion, while also following general PCI guidelines for coronary artery disease. LVEF was calculated from echocardiography using the biplane Simpson method.

### Data collection and follow-up

Procedural and clinical outcome data were collected using a dedicated database from three medical-center registries. To ensure accurate assessment for clinical outcomes during the follow-up period, information was obtained from each hospital’s medical chart review or through telephone contact. Additional information was obtained from the National Population Registry of the Korean National Stationary Office with the use of a unique personal identification number. All outcomes of interest were adjudicated by board-certified cardiologists blinded to the study purpose. This study was approved by the Institutional Review Board of Korea University Anam Hospital (2019AN0041). The informed consent was waived. The study also complied with the Declaration of Helsinki.

### Statistical analyses

Baseline patient demographics were analyzed using Student’s *t* test for continuous variables and the Chi square test or Fisher’s exact test for categorical variables as appropriate. Changes between baseline SYNTAX score and residual SYNTAX score in each SCR group in the PLVSF group and the RLVSF group were analyzed using the paired *t* test, respectively. Continuous variables are presented as mean ± standard deviations. Categorical variables are expressed as counts (percentages).

Propensity score was calculated from each subject using the logistic regression model including baseline covariates in the model. Age and the variables that were significantly different between the classified 2 groups were selected to calculate the propensity score. Inverse probability of treatment weighting (IPTW) were computed from the propensity score to control for confounding and selection bias and to adjust for significant differences in baseline characteristics between the SCR and OMT groups. Weights for patients receiving SCR were the inverse of the propensity score, and weights for patients receiving OMT were the inverse of (1-propensity score). An absolute standardized mean difference of < 10% for the measured covariate after IPTW adjustment indicated an appropriate balance between the two groups.

Event rates were estimated using Kaplan–Meier survival analysis, and hazard ratios (HR) with a 95% confidence interval (CI) were generated using Cox regression analysis. To determine the association between clinical characteristics and outcomes, multivariate Cox regression analyses were performed for the entire population. Cox regression models with tests for interaction were used to evaluate the consistency of treatment effects in subgroups. Survival differences between SCR and OMT in the PLVSF and RLVSF groups were compared with an interaction test using contrast weights [8]. A two-sided *p* value < 0.05 was considered statistically significant. All statistical analyses were performed using SAS 9.4 (SAS Institute Inc., Cary, NC, USA).

## Results

### Patients and lesion characteristics

**Fig. 2:**
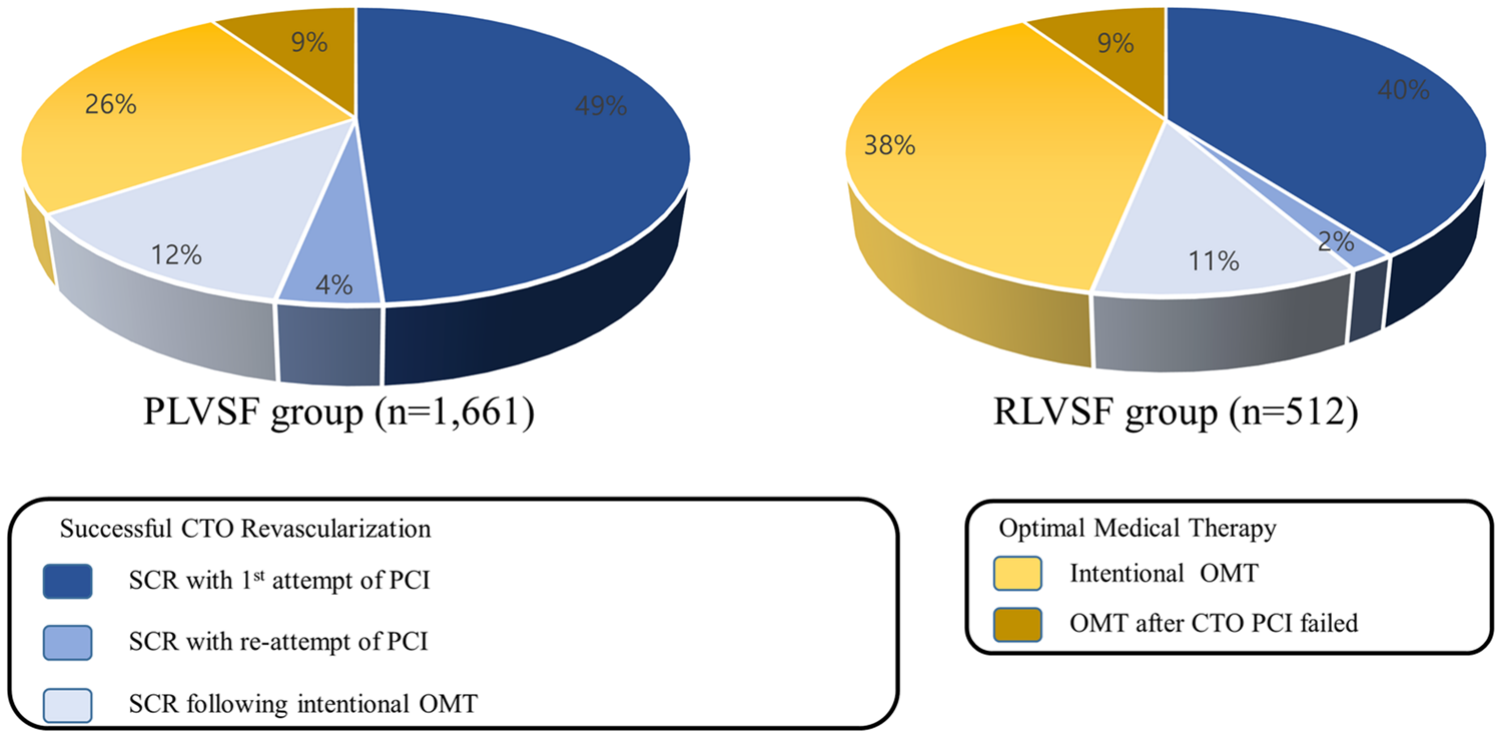
Distribution pattern differences in treatment assignment between patients with PLVSF and RLVSF. *CTO* chronic total occlusion; *OMT* optimal medical therapy; *PCI* percutaneous coronary intervention; *PLVSF* preserved left ventricular systolic function; *RLVSF* reduced left ventricular systolic function; *SCR* successful chronic total occlusion revascularization

Median follow-up duration of study population was 1138 days. Among a total of 2173 patients, 76.4% (*n* = 1661) had PLVSF (mean LVEF = 61%) and 23.6% (*n* = 512) had RLVSF (mean LVEF = 37%). Among the PLVSF patients, 1069 underwent SCR, and 592 were treated with OMT. Among the RLVSF patients, 272 underwent SCR, and 240 were treated with OMT (Fig. 1). OMT was usually the initial treatment of choice for CTO patients with RLVSF compared with PLVSF patients. When initial CTO-PCI failed, re-attempts were fewer in RLVSF patients than PLVSF patients. Consequently, OMT was more frequently selected as the final therapy option for CTO patients with RLVSF than with PLVSF (Fig. 2).

Baseline clinical and angiographic characteristics are summarized in Table 1. In patients with PLVSF, patients treated with SCR were younger (62.29 ± 11.18 vs. 64.07 ± 10.85 years, *p* = 0.0018), had a higher multi-vessel disease incidence (70.64% vs. 65.01%, *p* = 0.0186), and had a higher incidence of CTO lesions in the left anterior descending coronary artery (36.67% vs. 25.34%, *p* < 0.0001). Conversely, patients treated with OMT had lower SYNTAX scores (19.62 ± 8.61 vs. 17.34 ± 9.02, *p* < 0.0001) and histories of more frequent stroke, MI, and PCI. Patients treated with OMT tended to have higher J-CTO scores than SCR patients. The SYNTAX score decreased significantly after PCI in the SCR patients (Baseline 19.62 ± 8.61, Residual 6.23 ± 7.51, *p* < 0.001).

**Table 1:** Baseline clinical and angiographical characteristics of the study population

	PLVSF group (*n* = 1661)			RLVSF group (*n* = 512)		
	SCR (*n* = 1069)	OMT (*n* = 592)	*P* value	SCR (*n* = 272)	OMT (*n* = 240)	*P* value
Age (year)	62.29 ± 11.18	64.07 ± 10.85	0.0018	64.65 ± 11.43	68 ± 12.01	0.0013
EF (%)	61.44 ± 7.24	61.10 ± 7.45	0.3657	38.17 ± 8.01	36.31 ± 9.55	0.0183
Male gender	825 (77.17)	438 (73.99)	0.1448	213 (78.31)	184 (76.67)	0.65
Hypertension	686 (64.17)	399 (67.4)	0.18	160 (58.82)	150 (63.75)	0.25
Diabetes mellitus	430 (40.22)	246 (41.55)	0.5973	130 (47.79)	127 (52.92)	0.2473
Dyslipidemia	461 (43.12)	267 (45.1)	0.4367	91 (37.92)	111 (40.81)	0.504
Smoking	319 (29.84)	167 (28.21)	0.4839	86 (32.72)	65 (27.08)	0.1651
CKD	55 (5.14)	32 (5.41)	0.8195	31 (11.4)	38 (15.83)	0.1424
Previous stroke	66 (6.18)	65 (10.98)	0.0005	29 (10.66)	18 (7.5)	0.2163
Previous MI	146 (13.66)	116 (19.59)	0.0015	111 (40.81)	98 (40.83)	0.9915
Previous PCI	228 (21.33)	195 (32.94)	< 0.0001	68 (25)	71 (29.58)	0.2445
Previous ICD	–	–	–	–	–	–
Medications						
Aspirin	947 (88.59)	479 (80.91)	< 0.0001	240 (88.24)	164 (68.33)	< 0.0001
DAPT	1,011 (94.57)	499 (84.29)	< 0.0001	252 (92.65)	173 (72.08)	< 0.0001
Statin	794 (74.41)	389 (65.82)	0.0002	191 (70.48)	134 (56.3)	0.0009
Beta blocker	616 (58.72)	346 (59.15)	0.8678	175 (66.29)	153 (64.83)	0.732
ACEI	198 (19.24)	104 (18.18)	0.6033	91(35.27)	78 (33.62)	0.7011
ARB	397 (38.58)	241 (41.91)	0.191	110 (42.31)	80 (35.09)	0.1027
Multi-vessel disease	746 (70.64)	379 (65.01)	0.0186	203(75.75)	179 (74.9)	0.8244
CTO lesion						
LAD	392 (36.67)	150 (25.34)	< 0.0001	127 (46.69)	83 (34.58)	0.0054
LCX	317 (29.65)	189 (31.93)	0.3353	78 (28.68)	88 (36.67)	0.0539
RCA	471 (44.06)	315 (53.21)	0.0003	120 (44.12)	131 (54.58)	0.0181
Multivessel	116 (10.85)	64 (10.81)	0.9797	58 (21.32)	58 (24.17)	0.4431
Revascularization						
Balloon only	151 (14.13)	–	–	35 (12.87)	–	–
Stent	918 (85.87)	–	–	237 (87.13)	–	–
Stent type						
BMS	–	–	–	1/237 (0.42)	–	–
1st gen DES	430/918 (46.82)	–	–	99/237 (41.77)	–	–
2nd gen DES	476/918 (51.85)	–	–	128/237 (54.01)	–	–
BVS	12/918 (1.31)	–	–	9/237 (3.80)	–	–
Number of stents						
1	583/918 (63.51)	–	–	141/237 (59.49)	–	–
2	282/918 (30.70)	–	–	80/237 (33.76)	–	–
3 ≤	53/918 (5.77)	–	–	16/237 (6.75)	–	–
Post-PCI TIMI 3	1,038 (97.10)	–	–	262 (96.32)	–	–
SYNTAX score						
Baseline	19.62 ± 8.61	17.34 ± 9.02	< 0.0001	23.38 ± 9.4	21.43 ± 10.42	0.0723
Residual	6.23 ± 7.51	–	–	8.93 ± 9.14	–	–
* P* value^a^	< 0.001	–	–	< 0.001	–	–
J-CTO score	2.13 ± 1.11	2.26 ± 1.18	0.0848	2.12 ± 1.10	2.23 ± 1.17	0.0917

Data are mean ± standard deviation or number (%)

*ACEI* angiotensin converting enzyme inhibitor; *ARB* angiotensin II receptor blocker; *BMS* bare metal stent; *BVS* bioresorbable vascular scaffold; *CKD* chronic kidney disease; *CTO* chronic total occlusion; *DAPT* dual antiplatelet therapy; *DES* drug-eluting stent; *EF* ejection fraction; *ICD* implantable cardioverter defibrillator; *LAD* left anterior descending; *LCX* left circumflex; *MI* myocardial infarction; *OMT* optimal medical therapy; *PCI* percutaneous coronary intervention; *PLVSF* preserved left ventricular systolic function; *RCA* right coronary artery; *RLVSF* reduced left ventricular systolic function; *SCR* = successful chronic total occlusion revascularization; *TIMI* = Thrombolysis In Myocardial Infarction

^a^The *P* value was analyzed by the paired *t* test for the amount of change in the residual SYNTAX score compared to the baseline SYNTAX score of the SCR group

In patients with RLVSF, 18.1% had LVEF < 30%. Patients treated with SCR were younger (64.65 ± 11.43 vs. 68 ± 12.01 years, *p* = 0.0013) and had slightly higher LVEF and lower J-CTO scores than patients treated with OMT. Multivessel disease incidence was not different between patients treated with SCR and OMT. Similar to the PLVSF group patients, the SYNTAX score of SCR patients in the RLVSF group also decreased significantly after PCI (Baseline 23.38 ± 9.4, Residual 8.93 ± 9.14, *p* < 0.001).

Antiplatelet agents and statins were more frequently prescribed for patients treated with SCR, irrespective of LVSF, but prescription rates for beta blockers, calcium channel blockers, angiotensin converting enzyme inhibitors, and angiotensin II receptor blockers were similar between patients treated with SCR and OMT. The ratio of using either angiotensin converting enzyme inhibitors or angiotensin II receptor blockers was higher in the RLVSF group than in the PLVSF group. Different baseline demographics between patients treated with SCR and OMT in the PLVSF and RLVSF groups were adjusted for using IPTW for endpoint analysis (Online Resource 1).

### Clinical outcomes between CTO patients with PLVSF and RLVSF according to treatment assignment

**Fig. 3:**
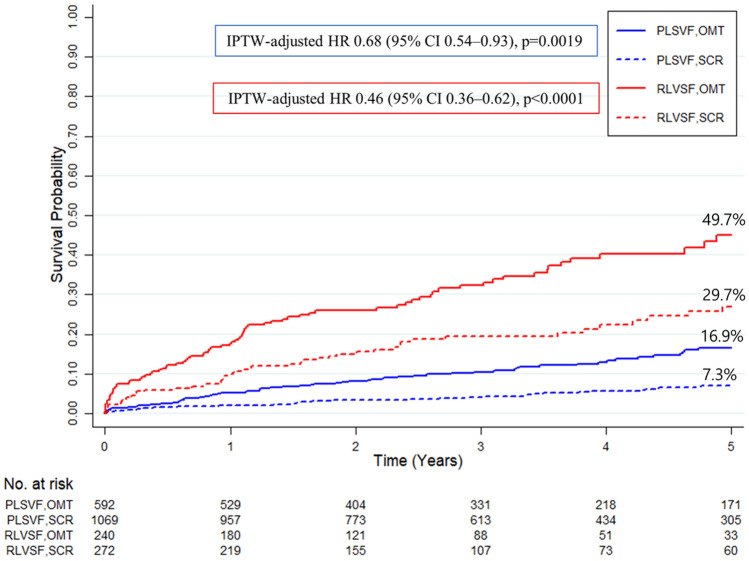
Kaplan–Meier analysis of the cumulative incidence of primary endpoint according to treatment assignment in CTO patients with PLVSF and RLVSF. The panel represents the time-to-event Kaplan–Meier curves for the cumulative incidence of composite all-cause death or non-fatal MI in CTO patients with PLVSF and RLVSF, according to treatment assignment. Results were compared with an IPTW method to adjust for baseline covariates. *CTO* chronic total occlusion; *HR* hazard ratio; *IPTW* inverse probability of treatment weighting; *MI* myocardial infarction; *OMT* optimal medical therapy; *PLVSF* preserved left ventricular systolic function; *RLVSF* reduced left ventricular systolic function; *SCR* successful chronic total occlusion revascularization

**Fig. 4:**
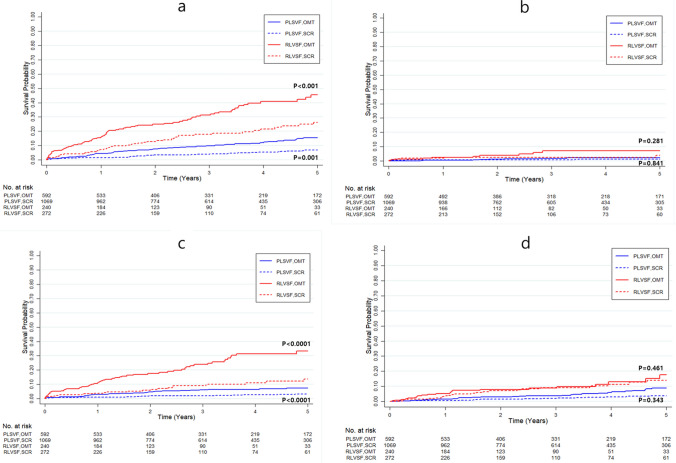
Kaplan–Meier analysis of the cumulative incidence of clinical outcomes according to treatment assignment in CTO patients with PLVSF and RLVSF. Panel **a** represents the time-to-event Kaplan–Meier curves for the cumulative incidence of all-cause death in CTO patients with PLVSF and RLVSF, according to treatment assignment. Panel **b** represents the cumulative incidence of non-fatal MI, according to treatment assignment. Panel **c** represents the cumulative incidence of cardiac death, according to treatment assignment. Panel **d** represents the cumulative incidence of non-cardiac death, according to treatment assignment. For all Panels (**a**–**d**), results were compared with an IPTW method to adjust for baseline covariates. *CTO* chronic total occlusion; *IPTW* inverse probability of treatment weighting; *MI* myocardial infarction; *OMT* optimal medical therapy; *PLVSF* preserved left ventricular systolic function; *RLVSF* reduced left ventricular systolic function; *SCR* successful chronic total occlusion revascularization

Clinical outcomes and IPTW-adjusted HRs for each endpoint are summarized in Table 2. In patients with PLVSF, the primary endpoint, composite all-cause death or non-fatal MI, occurred significantly less frequently in patients treated with SCR than with OMT (7.3% vs. 16.9%; HR 0.68; 95% CI 0.54–0.93; *p* = 0.0019; Fig. 3). The difference was mainly driven by a reduction in cardiac death (4.2% vs. 11.4%; HR 0.45; 95% CI 0.30–0.66; *p* < 0.0001), whereas incidences of MI and non-cardiac death were not different between patients treated with SCR and OMT (Fig. 4).

**Table 2:** IPTW-adjusted hazard ratios for clinical outcomes

	SCR	OMT	IPTW†-adjusted	
	Events rates‡	Events rates	HR (95% CI)	*P* value
PLVSF group	*n* = 1069	*n* = 592		
All-cause death or non-fatal MI	52 (7.3%)	70 (16.9%)	0.68 (0.54–0.93)	0.0019
All-cause death	49 (7.3%)	65 (16.7%)	0.65 (0.54–0.92)	0.0010
Cardiac death	24 (4.2%)	34 (11.4%)	0.45 (0.30–0.66)	< 0.0001
MI	11 (1.0%)	9 (1.6%)	1.08 (0.51–2.26)	0.841
Non-cardiac death	25 (3.1%)	31 (5.3%)	0.88 (0.65–1.22)	0.461

RLVSF group	*n* = 272	*n* = 240		
All-cause death or non-fatal MI	51 (29.7%)	79 (49.7%)	0.46 (0.36–0.62)	< 0.0001
All-cause death	47 (29.6%)	78 (49.6%)	0.43 (0.48–0.86)	0.0009
Cardiac death	23 (19.2%)	54 (37.4%)	0.35 (0.22–0.49)	< 0.0001
MI	7 (2.6%)	10 (4.6%)	0.72 (0.41–1.30)	0.281
Non-cardiac death	23 (10.4%)	22 (12.3%)	0.87 (0.56–1.29)	0.343

Data are number (%)

*CI* confidence interval; *HR* hazard ratio; *IPTW* inverse probability of treatment weighting; *MI* myocardial infarction; *OMT* optimal medical therapy; *PLVSF* preserved left ventricular systolic function; *RLVSF* reduced left ventricular systolic function; *SCR* successful CTO revascularization

^†^Adjusted HR for SCR compared with OMT was calculated using weighted Cox proportional hazard models

^‡^Events rates were calculated based on Kaplan–Meier analysis of the cumulative incidence

Similar to results for patients with PLVSF, patients with RLVSF had a significantly lower incidence of primary endpoint after treatment with SCR than with OMT (29.7% vs. 49.7%; HR 0.46; 95% CI 0.36–0.62; *p* < 0.0001; Fig. 3). The difference was mainly driven by a reduction in cardiac death (19.2% vs. 37.4%; HR 0.35; 95% CI 0.22–0.49; *p* < 0.0001); non-cardiac death and MI incidence was not different between the two groups (Fig. 4). Number needed to treat to save one life within 5 years was five in patients with RLVSF (95% CI, 0.48–0.86) and 10.5 in patients with PLVSF (95% CI, 0.54–0.92).

**Fig. 5:**
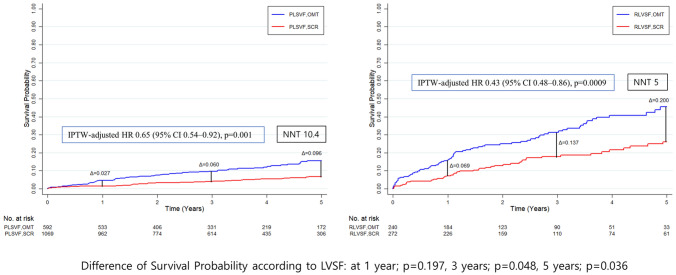
Difference in survival benefit of SCR between CTO patients with PLVSF and RLVSF. Cumulative incidence of all-cause death was compared according to treatment assignment, with an IPTW to adjust for baseline covariates. Survival probability differences with the use of a contrast test and NNT to save one life within 5 years were compared according to LVSF. The survival benefit from SCR between CTO patients with PLVSF and RLVSF was significantly different after three years and became more pronounced over time. *CI* confidence interval; *CTO* chronic total occlusion; *HR* hazard ratio; *IPTW* inverse probability of treatment weighting; *LVSF* left ventricular systolic function; *NNT* number needed to treat; *OMT* optimal medical therapy; *PLVSF* preserved left ventricular systolic function; *RLVSF* reduced left ventricular systolic function; *SCR* successful chronic total occlusion revascularization

The survival benefit from SCR was greater for CTO patients with RLVSF than with PLVSF and increased significantly over time (*p* = 0.197, *p* = 0.048, and *p* = 0.036 at 1, 3, and 5 year, respectively; Fig. 5).

### Interaction between treatment assignment and various subgroups

**Fig. 6:**
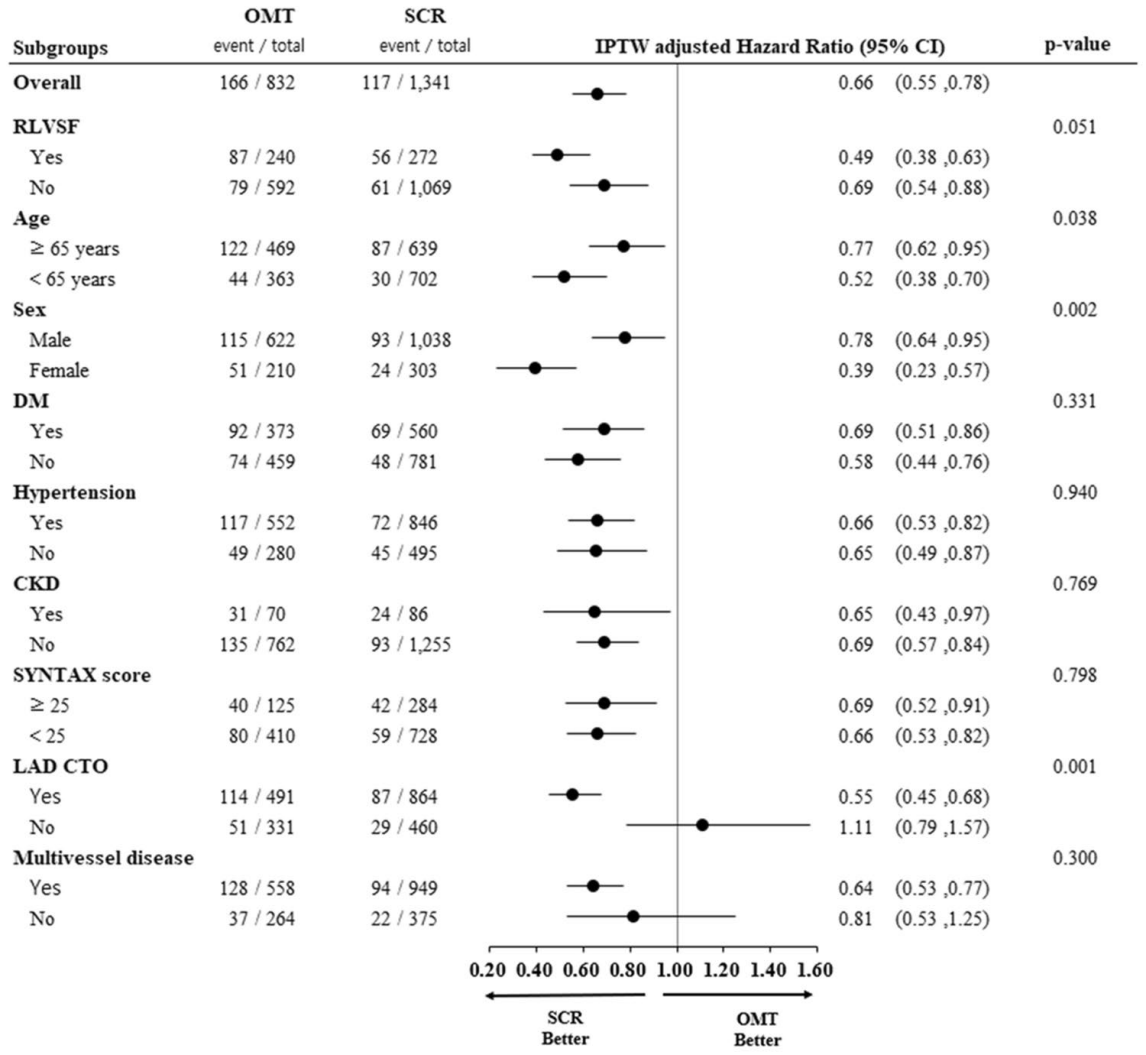
Interaction between treatment assignment and various subgroups for primary endpoint. Shown are the results of a Cox proportion-hazards model that tested for an interaction between various subgroups and treatment assignment, with an IPTW to adjust for baseline covariates. *CI* confidence interval; *CKD* chronic kidney disease; *CTO* chronic total occlusion; *DM* diabetes mellitus; *IPTW* inverse probability of treatment weighting; *LAD* left anterior descending; *OMT* optimal medical therapy; *RLVSF* reduced left ventricular systolic function; *SCR* successful chronic total occlusion revascularization

Although SCR was associated with a lower incidence of composite all-cause death or non-fatal MI than OMT, the interaction between the various subgroups and the beneficial effect of SCR over OMT was not significant, with the exception of the non-left anterior descending CTO subgroup (Fig. 6). The survival benefit of SCR was consistently observed in most subgroups.

### Independent predictors of the primary endpoint for CTO patients

Online Resource 2 shows independent predictors of the primary endpoint based on IPTW-adjusted multivariable Cox regression analysis. In the overall study population, RLVSF (HR 3.285; 95% CI 2.753–3.919) and chronic kidney disease (HR 3.824; 95% CI 3.029–4.826) were strong independent risk factors for the primary endpoint, and SCR was an independent protective factor (HR 0.579; 95% CI 0.486–0.691) in both groups.

### Clinical impact of CTO revascularization according to coronary anatomical features of CTO

Online Resource 3 shows the comparison of prognosis regarding primary endpoint according to the coronary anatomical location of CTO lesions of SCR patients in the PLVSF group and the RLVSF groups, respectively. As a result of the IPTW-corrected analysis, there was no difference in the prognosis according to the location of the coronary artery in the PLVSF group (panel a), but the prognosis of the left anterior descending CTO (HR 2.748; 95% CI 1.112–6.794; *p* = 0.0286) and the left circumflex CTO (HR 3.873; 95% CI 1.537–9.764; *p* = 0.0041) were worse when compared with the right coronary artery CTO in the RLVSF group, respectively (panel b).

## Discussion

In this study, 2,173 CTO patients who were treated with either SCR or OMT and the differential prognostic effects of SCR over OMT according to LVSF status were analyzed. The major findings of this study are as follows: First, SCR yielded a significantly lower risk of composite all-cause death and non-fatal MI than OMT, regardless of LVSF status, mainly driven by a reduction in cardiac death. Second, the survival benefit of SCR was greater in patients with RLVSF than PLVSF, and the difference of survival benefit of SCR over OMT between the both groups increased significantly over time. Third, in the whole study population, a lower incidence of the primary endpoint in SCR-treated patients, compared with OMT-treated patients, was consistently observed among various subgroups except for the non-left anterior descending CTO subgroup. These findings indicate that patients with RLVSF should be actively treated with CTO revascularization because they might experience a greater benefit than patients with PLVSF. Fourth, among patients with SCR in the RLVSF group, CTO lesions of left anterior descending and left circumflex CTO showed poor prognosis than right coronary artery CTO, of which the left circumflex CTO had the worst prognosis. The complex anatomical features of left circumflex, such as poorer collaterals and limiting use of retrograde may have affected prognosis, as reported in previous study [9].

### Debate over CTO revascularization for future clinical outcomes

Several mechanisms have been proposed to explain why patients who receive SCR experience greater survival benefits than patients who receive OMT. Sudden cardiac death incidence in OMT patients was reported to be five times greater (2.7% vs. 0.5%) than in SCR patients [10]. Several studies found that SCR improved LVEF and reduced the predisposition to ventricular arrhythmia [11–13]. Another possible explanation is that, in the context of MI, SCR offers protection from possible sudden occlusion in other vessels that provide collateral for the CTO vessel [14].

However, in recent randomized controlled trials, the DECISION-CTO (Drug-Eluting stent Implantation versus optimal Medical Treatment in patients with ChronIc Total OccluSION), EUROCTO (A Randomized Multicentre Trial to Evaluate the Utilization of Revascularization or Optimal Medical Therapy for the Treatment of Chronic Total Coronary Occlusions), and EXPLORE (Evaluating Xience and Left Ventricular Function in Percutaneous Coronary Intervention on Occlusions After ST-Elevation Myocardial Infarction) trials, a significant survival benefit of CTO-PCI was not found [15–17]. One possible explanation for the negative results is that PCI was implemented without considering risk severity based on the ischemic burden associated with CTO lesions. The effects of CTO revascularization between low-risk CTO lesions with mild ischemia and high-risk CTO lesions with moderate-to-severe ischemia are obviously different.

### Effects of SCR in patients with RLVSF versus PLVSF

To date, data on CTO patients with RLVSF are limited because most CTO-related clinical studies excluded patients with RLVSF. Therefore, the number of patients with RLVSF among all CTO patients and their clinical outcomes after SCR are unknown.

RLVSF with CTO may represent a larger ischemic burden caused by CTO and relevant donor vessels than PLVSF if a considerable amount of myocardium is viable. Therefore, CTO patients with RLVSF who are found to have sufficient viable myocardium might receive greater benefit from SCR than patients with PLVSF.

Myocardial viability tests have been proposed as a key factor in the decision-making process concerning coronary revascularization procedures for ischemic heart failure patients, based on observational studies [18, 19]. However, these studies were limited by retrospective design, probable selection biases for revascularization, and inadequate adjustment for baseline comorbidities.

Although the present study did not include a myocardial viability test and did not demonstrate an improvement in LVEF after SCR, the survival benefit from SCR was much greater in patients with RLVSF than with PLVSF. Therefore, SCR should be actively promoted as a treatment option and the decision to pursue PCI for CTO revascularization should not be done solely on the basis of viability test results for patients with RLVSF. Given recent report that similar success rates and safety in elderly and young CTO patients might be achieved, SCR through PCI would be a preferred treatment option instead of coronary artery bypass graft especially for elder CTO patients with RLVSF [20].

## Limitations

First, this study had inherent limitations associated with a retrospective study design. Although unbalanced factors were corrected using IPTW, completely eliminating effects of hidden factors on clinical outcomes was not possible. Second, because a viability test was not performed in patients with RLVSF, analysis of clinical outcome differences based on viability testing was not possible. Third, since two-dimensional echocardiography was not regularly performed in patients with RLVSF, analyzing clinical outcomes based on LVSF improvement was not available. Fourth, because patients with finally failed CTO-PCI were assigned to the OMT group, the OMT group might have had the worse results due to unfavorable influence of these particular patient subgroup. Also, OMT was not protocolized and left to the decision of the attending physician in present study. Medical and interventional treatment after the successful or failed CTO recanalization were inevitably different and this might interact with the endpoints of interest in this study. However, these finally failed CTO-PCI patients were assigned at a relatively similar rate to the PLVSF and RLVSF groups. Therefore, the negative effect on clinical outcomes of OMT between the PLSVF group and the RLVSF group is thought to be similar. Fifth, it was unable to assess the impact of implantable cardioverter defibrillator on clinical outcomes in the RLVSF group. Implantable cardioverter defibrillator implantation was not recommended for primary prevention in patients with LVEF < 35% in Korea at the time when the subjects of this study were collected. Sixth, we are lacking data on CTO crossing strategies, which might influence to clinical outcome.

## Conclusion

SCR was associated with better survival benefit than OMT, mainly driven by a reduction in cardiac death in both PLVSF and RLVSF groups. The benefits were greater especially in patients with RLVSF than in those with PLVSF. The difference in survival benefit from SCR between PLVSF and RLVSF patients increased over time.

## Electronic supplementary material

Below is the link to the electronic supplementary material.

Supplementary file1 (PDF 261 kb)

## Data Availability

Not applicable.
