# Supplementary material for: Differential clinical impact of chronic total occlusion revascularization based on left ventricular systolic function
Source: Clin Res Cardiol. 2020 Sep 2;110(2):237–48. doi: 10.1007/s00392-020-01738-2 (PMC7862506; doi:10.1007/s00392-020-01738-2)
Supplement: Supplementary file 1 — Supplementary file1 (PDF 261 kb) [file 392_2020_1738_MOESM1_ESM.pdf]

Differential clinical impact of chronic total occlusion revascularization based on left ventricular systolic function

Clinical Research in Cardiology

Hyungdon Kook, MD[a*]; Jeong Hoon Yang, MD [b*]; Jae Young Cho, MD [c]; Duck Hyun Jang, MD [a]; Min Sun Kim, MS [d]; Juneyoung Lee, PhD [d]; Seung Hun Lee, MD [e]; Hyung Joon Joo, MD [a]; Jae Hyoung Park, MD [a]; Soon Jun Hong, MD [a]; Je Sang Kim, MD [e]; Hyun Jong Lee, MD [e]; Rak Kyeong Choi, MD [e]; Young Jin Choi, MD [e]; Jin Sik Park, MD [e]; Young Bin Song, MD [b]; Jin-Ho Choi, MD [b]; Joo-Yong Hahn, MD [b]; Hyeon-Cheol Gwon, MD [b]; Do-Sun Lim, MD [a]; Seung-Hyuk Choi, MD [b]; Cheol Woong Yu, MD [a]

*The first two authors contributed equally to this article.

The last two authors are co-corresponding authors.

[a]Division of Cardiology, Department of Internal Medicine, Korea University Anam Hospital, Korea University College of Medicine, Seoul, Korea; [b]Division of Cardiology, Department of Internal Medicine, Samsung Medical Center, Sungkyunkwan University College of Medicine, Seoul, Korea; [c]Department of Cardiovascular Medicine, Regional Cardiocerebrovascular Center, Wonkwang University Hospital, Iksan, Korea; [d]Department of Biostatistics, Korea University College of Medicine, Seoul, Korea; [e]Division of Cardiology, Department of Internal Medicine, Sejong General Hospital, Bucheon, Korea

Address for correspondence:

Cheol Woong Yu, MD, PhD

Cardiovascular Center, Anam Hospital, Korea University Medical Center,

#73, Goryeodae-ro, Sungbuk-ku, Seoul, 02841, Korea

E-mail: ycw717@naver.com

Seung-Hyuk Choi, MD, PhD

Division of Cardiology, Department of Medicine, Samsung Medical Center, Sungkyunkwan University School of Medicine, Seoul, Korea

#81, Irwon-ro, Gangnam-gu, Seoul. 06351, Korea

E-mail: cardiochoi@skku.edu

Electronic Supplementary Material

Online Resource 1. IPTW adjusted Baseline clinical characteristics of the study population

	PLVSF group					RLVSF group				
	SCR		OMT		P value	SCR		OMT		P value
Age (yr)	63.22	±13.73	63.09	±17.53	0.8297	65.68	±14.34	64.03	±19.70	0.2021
EF (%)	61	±7.94	60.97	±12.24	0.9316	37.3	±10.21	37.81	±13.72	0.578
Male gender	927	(76.99)	997	(87.12)	0.4983	258	(80.58)	311	(82.45)	0.5257
Hypertension	783	(65.02)	826	(64.76)	0.8919	222	(69.21)	237	(62.91)	0.0803
Diabetes mellitus	481	(39.94)	507	(39.76)	0.9301	183	(57.11)	224	(59.51)	0.5227
Dyslipidemia	341	(28.33)	369	(28.90)	0.7516	77	(24.05)	97	(25.85)	0.5834
Smoking	353	(29.35)	309	(24.18)	0.0036	116	(36.26)	173	(45.99)	0.0094
Previous CKD	81.35	(6.76)	106	(8.28)	0.1514	41	(12.70)	52	(13.92)	0.6369
Previous stroke	80	(6.67)	94	(7.34)	0.5145	50	(15.74)	35	(9.38)	0.0109
Previous MI	201	(16.69)	225	(17.60)	0.5476	157	(48.92)	235	(62.41)	0.0003
Previous PCI	310	(25.78)	311	(24.37)	0.4179	108	(33.70)	115	(41.15)	0.0431

ACS	225 (18.69)	246 (19.31)	0.6974	93 (29.09)	130 (34.57)	0.1223

Data are mean ± standard deviation or number (%). ACS=acute coronary syndrome; CKD=chronic kidney disease; EF=ejection fraction; IPTW=inverse probability of treatment weighting; MI=myocardial infarction; OMT=optimal medical therapy; PCI=percutaneous coronary intervention; PLVSF=preserved left ventricular systolic function; RLVSF=reduced left ventricular systolic function; SCR=successful chronic total occlusion revascularization.

Online Resource 2. Multivariate Cox Regression Analysis for Independent Predictor of Primary Endpoint in Overall CTO Patients

	Overall			PLVSF group			RLVSF group		
	HR	95% CI		HR	95% CI		HR	95% CI	
RLVSF	3.285	2.753	3.919	NA	NA	NA	NA	NA	NA
SCR	0.579	0.486	0.691	0.64	0.5	0.819	0.455	0.349	0.594
Sex	0.871	0.704	1.078	0.936	0.702	1.248	0.851	0.603	1.2
Age (per year)	1.053	1.044	1.063	1.072	1.057	1.088	1.041	1.028	1.054
Smoking	1.534	1.252	1.879	0.768	0.555	1.062	2.554	1.886	3.459
DM	1.428	1.194	1.707	1.677	1.312	2.144	1.085	0.827	1.424
Hypertension	0.708	0.586	0.855	0.604	0.456	0.799	0.737	0.557	0.975
Dyslipidemia	0.635	0.512	0.787	0.284	0.193	0.418	1.209	0.908	1.609
CKD	3.824	3.029	4.826	4.345	3.061	6.168	3.426	2.457	4.777
CTO lesion									
LAD	1.09	0.89	1.334	1.267	0.96	1.672	1.1	0.811	1.493
LCX	1.387	1.138	1.69	1.568	1.203	2.045	1.621	1.177	2.231
RCA	1.04	0.852	1.27	1.369	1.026	1.825	0.899	0.667	1.212

CI=confidence interval; CKD=chronic kidney disease; CTO=chronic total occlusion; DM=diabetes mellitus; HR=hazard ratio; LAD=left anterior descending; LCX=left circumflex; PLVSF=preserved left ventricular systolic function; RCA=right coronary artery; RLVSF=reduced left ventricular systolic function; SCR=successful CTO revascularization.

Online Resource 3. Comparison of primary endpoint according to the coronary anatomical location of CTO lesions of SCR patients

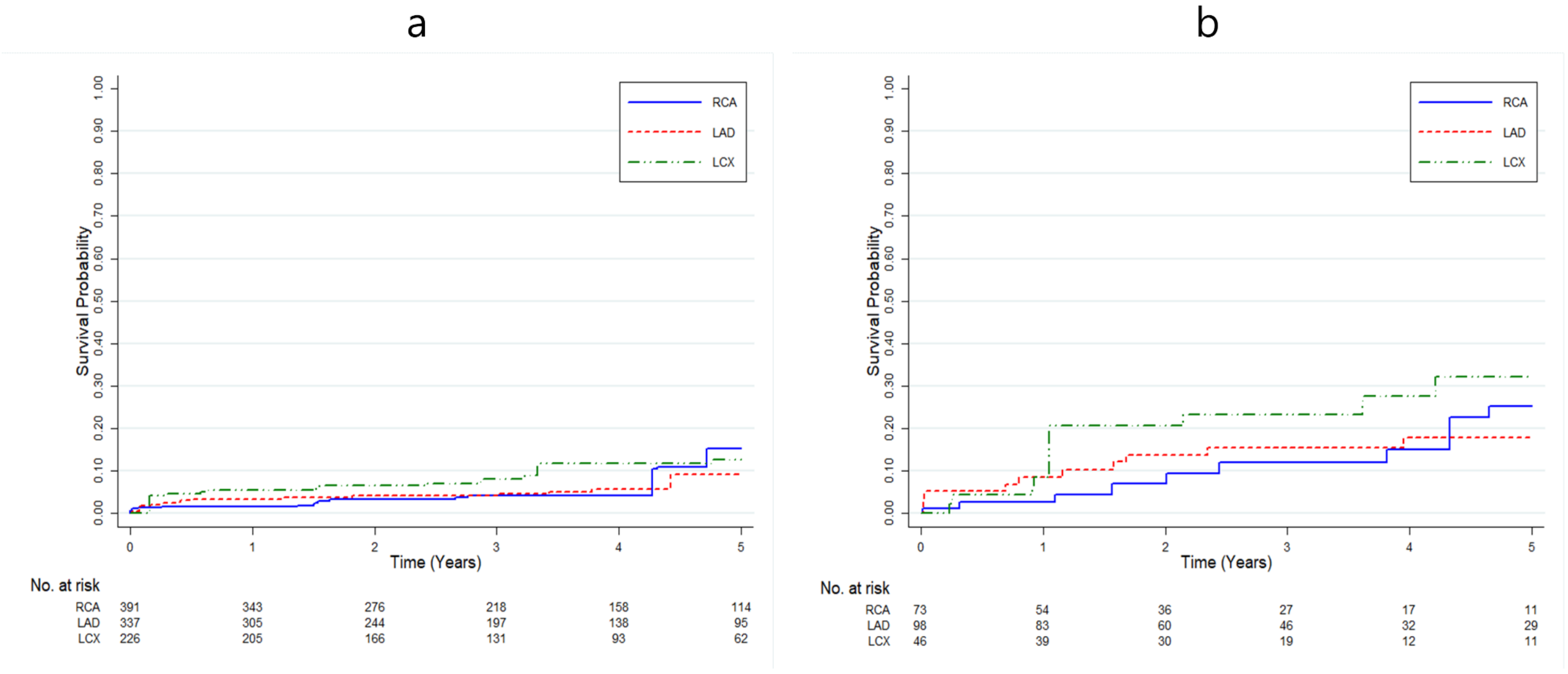

Panel a represents comparison according to the coronary anatomical location of CTO lesions of SCR patients in the PVLSF group. Panel b

represents comparison according to the coronary anatomical location of CTO lesions of SCR patients in the RVLSF group. CTO=chronic total occlusion; LAD=left anterior descending; LCX=left circumflex; PLVSF=preserved left ventricular systolic function; RCA=right coronary artery; RLVSF=reduced left ventricular systolic function; SCR=successful CTO revascularization
